# Mechanisms of the Effects of Polyphenols on Diabetic Nephropathy

**DOI:** 10.3390/cimb47090735

**Published:** 2025-09-10

**Authors:** Masumi Kamiyama, Kotoe Iijima, Rema Okuzawa, Ruka Kawata, Airi Kimura, Yuki Shinohara, Ayana Shimada, Mika Yamanaka, Ayuka Youda, Tamami Iwamoto

**Affiliations:** Department of Food and Nutrition, Jumonji University, 2-1-28, Sugasawa, Niiza 352-8510, Saitama, Japan

**Keywords:** angiotensinogen, diabetic nephropathy, isoflavone, polyphenol, renin–angiotensin system, resveratrol

## Abstract

Diabetic nephropathy is a major challenge in medicine. While a variety of mechanisms underlie the onset and progression of diabetic nephropathy, oxidative stress is critical because it promotes inflammation and creates a vicious cycle that induces podocyte injury, extracellular matrix accumulation, glomerulosclerosis, epithelial–mesenchymal transition, tubular atrophy, and proteinuria. There are various treatments for diabetic nephropathy, and each has its own limitations. Although the exact mechanisms by which polyphenols suppress diabetic nephropathy have not been elucidated, they may have antioxidant, anti-inflammatory, antifibrotic, and/or anti-apoptotic effects. They may also suppress endoplasmic reticulum stress and ameliorate mitochondrial dysfunction and dyslipidemia. Dietary polyphenols may be able to prevent the onset and slow the progression of diabetic nephropathy; they include resveratrol, quercetin, isoflavones, catechins, and anthocyanidins and have antioxidant, anti-inflammatory, antifibrotic, and anti-apoptotic effects through multiple molecular targets. Furthermore, they have shown few side effects. However, further research is needed to fully elucidate the molecular mechanisms by which polyphenols exert their effects and to clarify their optimal therapeutic use. In this review, we summarize reports published in the past five years regarding their effects on diabetic nephropathy and provide an overview of the potential of polyphenols.

## 1. Introduction

Diabetic nephropathy is a major healthcare challenge, developing in up to 50% of patients with diabetes. It is the leading cause of end-stage kidney disease, which requires treatment via dialysis or kidney transplantation, and is associated with significantly increased risks of cardiovascular morbidity and mortality [1].

Although the pathogenesis of diabetic nephropathy is complex and yet to be fully elucidated, chronic hyperglycemia and hypertension are key predisposing factors [2]. Oxidative stress is critical in the development and progression of diabetic nephropathy, because it promotes inflammation and creates a vicious cycle that involves podocyte injury, extracellular matrix accumulation, glomerular sclerosis, epithelial–mesenchymal transition (EMT), renal tubular atrophy, and proteinuria. Free radicals are molecules that are generated during various biological processes and are involved in physiological responses [3]. However, they are also potent regulators of many pathological pathways, including apoptosis, necrosis, and inflammation.

The use of dipeptidyl peptidase-4 (DPP-4) inhibitors increases the risk of hypoglycemia, but importantly, DPP-4 inhibitors reduce the hypoxia-induced upregulation of the pro-oxidant enzyme, nicotinamide adenine dinucleotide phosphate oxidase, in rat cardiomyocytes. Furthermore, saxagliptin prevents nicotinamide adenine dinucleotide phosphate oxidase-mediated uncoupling of endothelial nitric oxide synthase and attenuates vascular remodeling in diabetic mice, and vildagliptin restores the oxidant/antioxidant balance by increasing the superoxide dismutase activity and glutathione content in rats with testosterone-induced benign prostatic hyperplasia and cisplatin-induced neurotoxicity [4]. DPP-4 inhibitors have been shown to have beneficial effects in diabetic kidney disease independent of their glucose-lowering ability, mediated by their antifibrotic, anti-inflammatory, and anti-oxidative stress properties [5]. This study suggests a new mechanism for the suppression of diabetic nephropathy, and future studies are anticipated.

The inhibitory effects of polyphenols on the onset and progression of diabetic nephropathy have been modynamic, and inflammatory factors. Immune cells play a key role in promoting inflammation by releasing proinflammatory cytokines and chemokines, which significantly contribute to the development and progression of diabetic nephropathy. These immune responses exacerbate renal injury through immune cell infiltration into kidney tissue, leading to further damage. Heightened inflammation mediated by these cytokines and chemokines is closely associated with renal fibrotic changes and exacerbates diabetic nephropathy [6].

Furthermore, diabetic nephropathy is strongly influenced by disruption of metabolic signaling pathways, particularly those regulating glucose and lipid metabolism. Hyperglycemia induces the formation of advanced glycation end products, which, together with oxidative stress, activates inflammatory pathways that promote renal injury. This creates a vicious cycle in which the immune response and metabolic dysregulation continually interact, further accelerating the progression of diabetic nephropathy. In addition, multiple inflammatory pathways and mediators, including angiotensin II, are involved in the onset and progression of diabetic nephropathy [6].

The development of treatments for diabetic nephropathy continues. For example, sodium–glucose cotransporter 2 inhibitors reduce inflammation and oxidative stress and reduce the risks of the progression of type 2 diabetes and the development of kidney disease in such patients [7]. However, the overall therapeutic effects of the currently available treatments are insufficient.

In this review, we discuss the pathogenesis of diabetic nephropathy, particularly the roles of oxidative stress, angiotensin II, and inflammation. We also discuss developments in therapeutic strategies, including the use of polyphenols and, especially, resveratrol, which target inflammation. Although the role of diet in diabetic nephropathy remains to be determined, it is thought that the efficacy, dose, and mechanism of effects of dietary antioxidants, particularly polyphenols, will become a subject of great interest in the future. Therefore, we summarize the effects of polyphenols that have been reported to date.

## 2. Chemistry of Polyphenols

**Figure 1:**
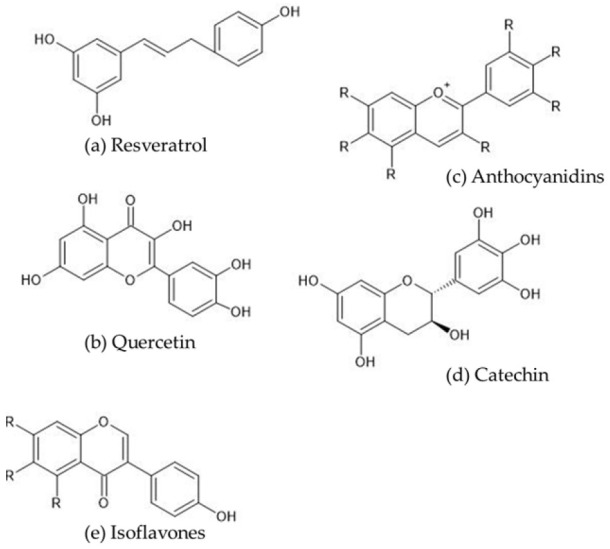
Chemical structures of selected polyphenols. (**a**): resveratrol (3,4′,5-trihydroxy-stilbene), (**b**): anthocyanidins (the chemical structure of anthocyanins includes polyhydroxy or polymethoxy derivatives of 2-phenylbenzophyllium), (**c**): quercetin (3,5,7,3′,4′-pentahydroxyflavone), (**d**): catechin (catechins comprise two benzene rings (A and B rings) linked by a dihydropyran heterocycle (C ring), which contains a hydroxyl moiety on carbon 3), (**e**): isoflavones (isoflavones are a group of compounds with a basic structure comprising two benzyl rings joined by a three-carbon bridge).

Polyphenols are compounds with multiple hydroxyl groups bound to a benzene ring (Figure 1), and, to date, over 8000 natural polyphenols have been identified [8]. They are generally classified as flavonoids, nonflavonoids, or phenolic acids [9]. Flavonoids can be divided into six subclasses: (i) flavonols (e.g., quercetin, kaempferol, myricetin, and isorhamnetin); (ii) flavones (e.g., luteolin and apigenin); (iii) isoflavones (e.g., daidzein and genistein); (iv) flavanones (e.g., naringenin and hesperidin); (v) flavanols (e.g., catechin, epicatechin, gallocatechin, epigallocatechin, and their gallates); and (vi) anthocyanidins (e.g., malvidin, cyanidin, delphinidin, and pelargonidin). The proanthocyanidins are conventionally considered to be condensed tannins. Nonflavonoids are further divided into three subclasses: stilbenoids, lignans, and diarylheptanoids, with resveratrol, curcumin, and coumarin being commonly studied examples. The phenolic acid class includes ellagic acid, tannic acid, gallic acid, caffeic acid, ferulic acid, syringic acid, and sinapic acid [10]. Although the exact mechanisms by which polyphenols suppress diabetic nephropathy have not been elucidated, antioxidant, anti-inflammatory, anti-fibrotic, and anti-apoptotic effects may be involved. They may also suppress endoplasmic reticulum stress and ameliorate mitochondrial dysfunction and dyslipidemia (Table 1) [11,12,13,14,15,16,17,18].

For example, when patients with diabetes drank green tea containing 800 mg of epigallocatechin gallate daily for 12 weeks, their urinary albumin-to-creatinine ratio decreased by 41%, implying that it had a renoprotective effect [56]. In addition, the administration of apple polyphenols to rats with streptozotocin (STZ)-induced diabetes for 10 weeks lowered their blood urea nitrogen-to-creatinine ratio and urinary albumin-to-creatinine ratio, reduced lipid peroxidation in their kidneys, and protected against diabetic nephropathy through an improvement in renal function and a reduction in oxidative stress [57]. Furthermore, it has been reported that the administration of naringin (50 mg/kg), chlorogenic acid (10 mg/kg), and quercetin (50 mg/kg) to rats with STZ-induced diabetes for 10 weeks ameliorated their renal injury and fibrosis by improving antioxidant status and reducing transforming growth factor-beta (TGF-β), tumor necrosis factor (TNFα), and p53 expression [58].

Mushrooms have also been reported to contain polyphenols, as well as a variety of bioactive compounds, including terpenoids, flavonoids, tannins, alkaloids, and polysaccharides, that contribute to their therapeutic effects against diabetes. Extracts from the fruiting bodies and mycelium of Ganoderma lucidum and Volvariella species have shown antioxidant and antimutagenic properties, suggesting their potential in the prevention and management of various diseases, including cancer. Their potent antioxidant activity helps neutralize harmful free radicals, which are unstable molecules that can harm cellular components such as DNA, lipids, and proteins and contribute to degenerative diseases such as cardiovascular and neurodegenerative diseases.

Below, we summarize the effects of selected polyphenols that have been reported over the last five years. The inhibitory effects of polyphenols on the onset and progression of diabetic nephropathy have been demonstrated. This review will introduce representative polyphenols, including resveratrol, quercetin, anthocyanidins, anthocyanins, catechin, and isoflavones. Their representative structures are shown in Figure 1. Detailed structures are explained in each section.

## 3. Renoprotective Effect of Resveratrol in Diabetic Nephropathy

The effects of the polyphenol resveratrol on diabetic nephropathy have been studied. Resveratrol (3,4′,5-trihydroxy-stilbene) is found in the skin and seeds of grapes and also in relatively large amounts in the traditional Chinese medicine ingredient *Polygonum cuspidatum* [59,60,61]. Resveratrol has diverse biological and pharmacological effects, including anti-obesity, antidiabetic, anticancer, anti-inflammatory, antioxidant, and cardiovascular protective properties [62]. Many in vivo studies have shown that resveratrol has glucose-lowering effects in type 1 and type 2 diabetic nephropathy [23,63,64]. In general, the principal aims of the treatment of diabetes are to reduce blood glucose concentrations, improve insulin sensitivity, and preserve pancreatic β cells, and resveratrol has been shown to have all of these effects [17,23,26,27,28,29,30,31,33].

We performed a literature search in PubMed using the terms “diabetic nephropathy”, “polyphenols”, and “resveratrol”. Combinations of resveratrol with other compounds have previously been evaluated for use in the treatment of diabetic nephropathy. For example, a combination of resveratrol (15 mg/kg/day) and ramipril (10 mg/kg/day) suppressed early glomerulosclerosis in rats with STZ-induced diabetes via the RhoA/ROCK pathway [19]. In addition, treatment of type 1 diabetes with a combination of resveratrol and human umbilical cord mesenchymal stem cells increased the number of podocytes and the expression of the podocyte-associated protein nephrin and Wilm’s tumor 1 (WT1), resulting in an amelioration of renal pathology [20]. Furthermore, administration of resveratrol and quercetin to aged rats reduced hyperglycemia-induced methylglyoxal levels and senescence marker protein 30 (SMP30) expression. Methicillin-resistant glycerol is a highly reactive α-ketoaldehyde that is produced as a byproduct of glycolysis. It can cause mitochondrial dysfunction and greater generation of reactive oxygen species, which increases oxidative stress. SMP30 expression decreases with age and is used as a marker of aging [65]. These results indicate that a combination of resveratrol and quercetin has antioxidant and anti-aging effects [28].

In rabbits with diabetic nephropathy, Resveratrol ameliorates renal hypoxia, mitochondrial dysfunction, and the apoptosis of renal tubular cells by activating the silent signaling regulator l (SIRT1), peroxisome proliferator-activated receptor gamma coactivator 1 alpha (PGC1α), and hypoxia-inducible transcription factor 1 alpha (HIF1α) signaling pathways [21]. It also reduces the expression of the proinflammatory proteins receptor for advanced glycation end products (RAGEs), NFκB p65, and NOX4, and consequently ameliorates renal pathology in non-obese diabetic mice [22]. Thus, resveratrol is thought to improve renal function not only through its anti-inflammatory effects but also by improving the metabolic memory of hyperglycemia. Resveratrol also improves lipid metabolism in diabetic nephropathy by participating in autophagy mediated by the AMP-activated protein kinase/mammalian target of the rapamycin (AMPKα/mTOR) pathway [22,23]. It also attenuates diabetic nephropathy in *db*/*db* mice by inhibiting oxidative stress-mediated apoptosis of podocytes, which is dependent on AMPK activation [24]. Finally, it protects renal tubular cells against hyperglycemia-induced apoptosis in diabetic nephropathy by suppressing endoplasmic reticulum stress [25]. In rat glomerular mesangial cells, resveratrol prevented or delayed the onset of lipopolysaccharide-induced proliferation and inflammatory mesangial cell-associated fibrosis, independent of its hypoglycemic effect [26]; in rat kidney cells treated with glyoxal, a dialdehyde consisting of two aldehyde groups (-CHO), resveratrol inhibited reactive oxygen species production and apoptosis [27].

C57BL/6J mice fed a high-fat diet for 12 weeks exhibited an increase in body weight and blood glucose and blood lipid concentrations, but resveratrol administration improved their circulating lipid and glucose concentrations, renal function, and the defective renal expression of the components of the JAML/SIRT1 lipid synthesis pathway and adipogenesis-related proteins [29]. In another study of high-fat diet-induced diabetes in mice, pterostilbene, a resveratrol analog, reduced the expression of TGFβ1, pSMAD3, SREBP1, and FAS, reduced ectopic lipid deposition, alleviated renal tubular injury and fibrosis, and improved renal function [30]. In addition, in mice with D-galactose-induced aging, resveratrol supplementation alleviated advanced glycation end product-related renal dysfunction by reducing renal cell senescence, apoptosis, and fibrosis [31]. Finally, resveratrol slowed the progression of diabetic nephropathy in *db*/*db* mice by preventing mitochondrial fission [17].

Polyphenols can also affect the expression of microRNAs (miRNAs), short (20–24 nucleotide) non-coding RNAs that regulate gene expression in multicellular organisms by influencing both mRNA stability and translation [66]. miRNAs are transcribed by RNA polymerase II as part of capped and polyadenylated primary transcripts (pri-miRNAs) that are either protein-coding or non-coding. The primary transcripts are cleaved by the Drosha ribonuclease III enzyme to generate approximately 70-nucleotide stem-loop precursor miRNAs (pre-miRNAs), which are further cleaved by the cytoplasmic Dicer ribonuclease to generate mature miRNA and antisense miRNA star (miRNA) products. Mature miRNAs are incorporated into the RNA-induced silencing complex (RISC), which recognizes target mRNAs through imperfect base pairing with miRNAs, most commonly resulting in the inhibition of translation or destabilization of the target mRNA species. In a high-glucose environment, resveratrol upregulates miR-1231, reduces the expression of insulin-like growth factor I (IGF1), inhibits the activity of the extracellular signal-regulated kinase (ERK) signaling pathway, and reduces the concentrations of the proinflammatory cytokines tumor necrosis factor alpha (TNFα) and interleukin 6 (IL6). Furthermore, an miR-1231 inhibitor weakens the protective effect of resveratrol on mesangial cells. Therefore, resveratrol has a protective effect on mesangial cells under high-glucose conditions, at least in part by regulating the miR-1231/IGF1/ERK pathway [32].

Finally, resveratrol has recently been shown to slow the progression of diabetic kidney disease in *db*/*db* mice by altering the composition of the gut microbiota and affecting the production of short-chain fatty acids, thereby suppressing tubulointerstitial fibrosis [33].

## 4. Renoprotective Effect of Quercetin in Diabetic Nephropathy

The exact mechanism by which quercetin ameliorates diabetic nephropathy has not been elucidated. However, previous studies have shown that it has antioxidant, anti-inflammatory, antifibrotic, and anti-apoptotic effects and that it can suppress endoplasmic reticulum stress and ameliorate mitochondrial dysfunction and dyslipidemia. Quercetin (3,5,7,3′,4′-pentahydroxyflavone) is found in vegetables such as onions, buckwheat, and fruit [10]. Following the administration of the equivalent of several grams of quercetin aglycone, quercetin aglycone and quercetin metabolites were found to be widely distributed in various tissues, at concentrations in the order of nanomoles per gram of tissue, with the highest concentrations being in the lung and the lowest concentrations in the brain and spleen [67]. Quercetin has anti-inflammatory, neuroprotective, and cardioprotective effects [68,69,70] and inhibits mesangial cell proliferation through the reactivation of the Hippo pathway in both the glomerular mesangium of high glucose-treated mice and in animal models of diabetic nephropathy [34]. We performed a literature search in PubMed using the terms “diabetic nephropathy” and “quercetin”. Quercetin also safely alleviates early diabetic kidney damage, possibly by improving lipid metabolism via the SREBP2 signaling pathway [35]. A study focusing on podocyte apoptosis demonstrated that quercetin attenuates podocyte apoptosis by inhibiting the EGFR signaling pathway, indicating that this pathway may represent a novel therapeutic target for diabetic nephropathy [36]. Quercetin also stimulates NRF2 and regulates the process of ferroptosis in *db*/*db* mice [38,39].

One study investigated the effects of a combination of atorvastatin and quercetin in NRK-52E rat kidney epithelial cells treated with the nephrotoxic agent STZ, and found that this combination significantly reduced the expression of TGFβ, TNFα, and IL6, suggesting that it has a renoprotective effect [40]. In addition, it has been reported that quercetin prevents diabetic nephropathy in STZ-induced type 2 diabetes by inhibiting the apoptosis of renal tubular epithelial cells via the PI3K/AKT pathway [41].

Quercetin suppresses hyperglycemia-induced proliferation, inflammation, and oxidative stress in human mesangial cells. The mechanism of this effect involves the direct binding of miR-485-5p to YAP1 to suppress YAP1 expression. The downregulation of miR-485-5p and upregulation of YAP1 were also identified in the serum of patients with diabetic nephropathy. Quercetin also inhibits HG-induced human mesangial cell proliferation, inflammation, and oxidative stress via the miR-485-5p/YAP1 axis, and therefore, this may provide a novel therapeutic target for diabetic nephropathy [37,71].

Quercetin also affects the expression of specific genes via a novel mechanism involving its binding to DNA or genome-associated proteins. Further exploration of its effects at the genome level and the genome-wide mapping of direct polyphenol–chromatin interactions may provide new insight into the mechanisms by which small molecules affect cell function, paving the way to a better understanding of responses to polyphenols in humans and the potential for predicting or controlling such responses [37].

Combinations of quercetin with drugs or other polyphenols may also ameliorate diabetic nephropathy. A combination of quercetin with dasatinib is the most studied senolytic drug combination and is used to treat various age-related diseases. Quercetin has been shown to significantly improve renal function, ameliorate histopathological and fibrotic changes, reduce lipid deposition, and increase the ATP concentrations of mice with diabetic kidney disease. Furthermore, it reduced the expression of multiple proteins involved in fatty acid oxidation and increased that of PPARα in *db*/*db* mice [72]. In diabetic kidney disease mice, increased expression of PPARα was confirmed to be effective in increasing the expression of downstream proteins involved in fatty acid oxidation, restoring renal function, and suppressing fibrosis in vitro and in vivo. Molecular docking and dynamics simulations also demonstrated that quercetin had renoprotective effects by binding to PPARα [42]. Other studies have shown that resveratrol and quercetin in herbal preparations act as powerful antioxidants and may ameliorate diabetic complications in the kidneys and prevent renal cell aging [28]. In combination with dasatinib and quercetin, binding to PPARα has a renoprotective effect, as shown by an amelioration of renal pathology and a reduction in extracellular matrix protein expression. It was also shown that the overexpression of PPARα has a renoprotective effect by upregulating the FAO pathway, which suggests that this may also be involved in the development of diabetic nephropathy [42].

## 5. Renoprotective Effect of Anthocyanidins and Anthocyanins in Diabetic Nephropathy

Anthocyanins are glycosylated forms of anthocyanidins. The flavylium cation skeleton is hydroxylated at various positions (commonly at carbons C3, C5, C6, C7, C3′, C4′, and C5′) to produce a variety of anthocyanidins. The chemical structure of anthocyanins includes polyhydroxy or polymethoxy derivatives of 2-phenylbenzophyllium. These phenolic compounds consist of two aromatic rings (A and B rings) linked by a three-carbon chain, forming an oxygenated heterocycle (C ring). Although the exact mechanisms by which anthocyanidins and anthocyanins ameliorate diabetic nephropathy have not been elucidated, they have antioxidant, anti-inflammatory, antifibrotic, and anti-apoptotic effects and ameliorate endoplasmic reticulum stress, mitochondrial dysfunction, and dyslipidemia [73].

We performed a literature search in PubMed using the terms “diabetic nephropathy”, “anthocyanidins”, or “anthocyanins” to identify relevant studies. Cyanidin-3-glucoside ameliorates diabetic nephropathy through effects on oxidative stress and inflammation exerted via the TGFβ1/SMAD2/3 pathway [74]. It also protects against high glucose-induced podocyte dysfunction; reduces autophagy by regulating the expression of LC3-II/LC3-I, Beclin1, and p62; and reduces apoptosis and EMT by activating the SIRT1/AMPK pathway [75].

A recent study revealed beneficial effects of anthocyanins on diabetic nephropathy through quantitative proteomic and nontargeted metabolomic analyses of kidney and serum samples, followed by bioinformatic analysis. Anthocyanins were shown to upregulate the taurine and hypotaurine metabolic pathways. In addition, the upregulation of tryptophan and tyrosine metabolism pathways was reduced in the serum of mice with diabetic nephropathy, indicating that the dysregulation of amino acid metabolism may be involved in diabetic nephropathy [76].

## 6. Renoprotective Effect of Catechin in Diabetic Nephropathy

Catechins comprise two benzene rings (A and B rings) linked by a dihydropyran heterocycle (C ring), which contains a hydroxyl moiety on carbon 3. The catechins are often classified as non-galloylated or galloylated, according to the absence or presence of a gallate moiety [77,78,79]. Although the exact mechanisms by which catechins ameliorate diabetic nephropathy have not been elucidated, they may have antioxidant, anti-inflammatory, antifibrotic, and anti-apoptotic effects, and they may reduce endoplasmic reticulum stress, mitochondrial dysfunction, and dyslipidemia.

We performed a literature search in PubMed using the terms “diabetic nephropathy” and “catechin”. (−)-Epigallocatechin gallate is one of the main bioactive components of green tea and has been shown to reduce renal fibrosis by targeting Notch via the inhibition of the TGFβ/SMAD3 pathway [53]. It has also been reported to attenuate renal fibrosis through reductions in the expression of fibronectin and Notch1 and the inhibition of the type II TGFβ receptor/Smad3 pathway in human embryonic kidney cells [53]. In diabetic kidneys, it was found that the administration of (−)-epigallocatechin gallate reduced Notch signaling and TGFβRII and Smad3 phosphorylation in the kidney. Thus, (−)-epigallocatechin gallate may ameliorate renal fibrosis by targeting Notch through the inhibition of the TGFβ/Smad3 pathway in diabetic mice [54].

In diabetic nephropathy, the expression of KIM1 is high, but (−)-epigallocatechin gallate intake has been shown to ameliorate hyperglycemia, hyperlipidemia, AGEs, renal pathological changes, renal dysfunction, and abnormal KIM1 protein expression. KIM-1 is a marker of obstruction-induced renal injury, and there is a new report showing that (-)-epigallocatechin gallate interacts with KIM-1 [80]. Diabetic nephropathy also involves NLRP3 inflammasome-induced inflammation, but supplementation with (−)-epigallocatechin gallate reduced the expression of ER stress markers and NLRP3 inflammasome components, implying that (−)-epigallocatechin gallate has anti-inflammatory effects that are principally exerted through the inhibition of endoplasmic reticulum stress and NLRP3. Further research into the potential for this molecule is underway [81].

Endoplasmic reticulum stress and chronic sterile inflammation have been implicated in the pathogenesis of diabetic nephropathy, and intriguingly, (+)-catechin has been reported to regulate endoplasmic reticulum stress and NLRP3-associated inflammation. (+)-Catechin significantly improves renal function and attenuates tubular injury by suppressing endoplasmic reticulum stress through reductions in the expression of GRP78, PEAK, CHOP, ATF6, and XBP1. (+)-Catechin also suppresses NLRP3-associated inflammation in the kidney, as demonstrated by reductions in NLRP3, ASC, AIM2, caspase 1, IL-1β, and IL-18 expression in mice with diabetic nephropathy and palmitic acid-treated HK-2 cells. Palmitic acid, a saturated fatty acid, has been reported to induce the expression of the apoptosis-related transcription factor CHOP through the activation of the PERK/ATF4 pathway. However, the mechanisms mediating the transmission of information regarding the changes in membrane lipid composition caused by fatty acids to the nucleus and their effects on the endoplasmic reticulum stress response have not been well characterized. (+)-Catechin has been suggested to have a renoprotective effect in DN by suppressing endoplasmic reticulum stress and NLRP3-related inflammation, thereby ameliorating tubular damage [55].

## 7. Renoprotective Effect of Isoflavones in Diabetic Nephropathy

Isoflavones are a group of compounds with a basic structure comprising two benzyl rings joined by a three-carbon bridge. This bridge may or may not be close to forming a pyran ring. Although the exact mechanisms by which isoflavones suppress diabetic nephropathy have not been elucidated, they have antioxidant, anti-inflammatory, antifibrotic, and anti-apoptotic effects, and they can also reduce endoplasmic reticulum stress, mitochondrial dysfunction, and dyslipidemia [82].

We performed a literature search in PubMed using the terms “diabetic nephropathy” and “isoflavones”. Formononetin is a polyphenolic phytoestrogen that is found in soybeans and other legumes and has been reported to have effects on SIRT1 and oxidative stress. In rats with STZ-induced diabetes that were fed a high-fat diet, the isoflavonoid formononetin reduced the high circulating concentrations of creatinine, urea nitrogen, and albumin [43]. Formononetin treatment has also been shown to enhance creatinine clearance, significantly reduce oxidative stress, and increase the renal expression of SIRT1, an anti-aging protein that is involved in diabetic nephropathy, in diabetic mouse and rat model animals [83]. SIRT1 negatively regulates the expression of p66, which reduces oxidative stress in the kidneys of SHC and protects against podocyte exhaustion [84]. It also reduces the level of hyperalbuminuria, fibrosis surrounding proximal tubule cells, and mesangial expansion in diabetic nephropathy [85,86]. SIRT1 regulates the renin–angiotensin system by activating the angiotensin-converting enzyme 2 (ACE2) promoter and causes vasodilation by decreasing the expression of angiotensin II receptor type I (AT1R). Moreover, it increases the production of nitric oxide, which increases the deacetylation of eNOS, which protects vascular tissue [84,87].

Calycosin, a bioactive phytoestrogenic isoflavone, reduced diabetes-induced inflammation in the kidney by suppressing the phosphorylation of IKBa and NFκB p65 in *db*/*db* mice [44,88]. The administration of tectorigenin, an O-methylated isoflavone and an ingredient of traditional Chinese medicines, to *db*/*db* mice ameliorated their abnormalities in glucose and lipid metabolism and reduced their renal inflammation, effects that were mediated by the activation of AdipoR1/2 [45].

Genistein administration to KK-Ay mice inhibited inflammatory responses and cell interactions in both mouse tubular cells and macrophages. It also suppressed the activation of inducible activator protein 1 and the development of oxidative stress, effects that were accompanied by a decrease in the expression of the gene encoding NADPH oxidase (NOX), in the presence of high glucose and albumin concentrations [46]. Furthermore, it reduced the levels of lipid peroxidation in both the plasma and urine and reduced Nox gene expression [46].

Genistein administration to Sprague Dawley rats was shown to improve their renal function by reducing their serum creatinine and blood glucose concentrations. The expression of NOX4, MAPK, p65, and p53 was downregulated, whereas the abnormal renal expression of MNF2 was reversed by genistein. Further investigations revealed that genistein suppressed the expansion of the mesangial matrix and oxidative stress, protected podocyte integrity, increased mitochondrial membrane potential, and improved mitochondrial function. Genistein may also alleviate diabetic nephropathy by inhibiting the MAPK/NFκB pathway and inhibiting inflammation [47]. A model of STZ-induced diabetes was shown to be characterized by high serum concentrations of IL1β and renal levels of NFκB p65, NLRP3, TXNIP, and MDA, but low levels of IL10 and TAC and Nrf2 gene expression. However, the levels of these biomarkers were significantly improved by calycosin treatment, which reduced NFκBp65/NLRP3/TXNIP signaling, oxidative stress, inflammatory cytokine levels, and fibrosis [48].

There have also been several reports on the effectiveness of puerarin against diabetic nephropathy. The administration of puerarin to a model of STZ-induced diabetes caused the inhibition of caspase 1-mediated pyroptosis [49], which is involved in the progression of diabetic nephropathy [89,90,91,92]. Pyroptosis has been described as a form of inflammatory programmed cell death and was first identified in macrophages infected with Salmonella typhimurium [93] or Shigella flexneri [94] in the 1990s. The death of these macrophages as a result of bacterial infection was found to be dependent on caspase 1 [95]. Subsequently, in 2001, pyroptosis was defined as a distinct form of apoptosis [96]. Pyroptotic cells have similar characteristics to apoptotic cells, such as caspase dependency, nuclear condensation, and DNA damage, but they differ from them by the presence of plasma membrane disruption, cell swelling, and osmotic lysis, which causes the release of all the cytoplasmic contents, including IL18, IL1β, and other proinflammatory cytokines [10]. Metabolic diseases such as diabetes are thought to be exacerbated by pyroptosis because it induces chronic inflammation associated with the production of cytokines that induce insulin resistance [97]. Additive renoprotective effects of puerarin and arctigenin, the main components of Fructus arctii, have previously been identified in *db*/*db* mice. We found that puerarin reduced p65 acetylation by activating SIRT1 and that arctigenin reduced NFκB p65 phosphorylation, possibly through the activation of PP2A. This is significant because the NFκB pathway is the principal inflammatory pathway in diabetic nephropathy [50]. In vitro experiments have demonstrated the antioxidant effects of isoflavones. For example, high glucose-treated renal mesangial cells demonstrated oxidative stress that was inhibited by puerarin via the RAGE/PKC/NOX4 axis [51].

SIRT1 expression was found to be low in mice with STZ-induced diabetes, but puerarin increased SIRT1 expression, inhibited the activation of the NLRP3/caspase 1 pathway, reduced podocyte pyroptosis, and alleviated their renal inflammatory injury. These findings indicate that puerarin inhibits podocyte pyroptosis and ameliorates podocyte damage and renal inflammatory damage via the SIRT1/NLRP3/caspase 1 pathway. It may also have an anti-pyroptotic effect by reducing oxidative stress [52].

## 8. Conclusions and Prospects

Polyphenols are natural plant extracts with various pharmacological effects. They can be extracted from a wide range of sources, are generally cheap, and are regarded as safe because of their dietary origin. In recent years, numerous effects of polyphenols on diabetic nephropathy have been reported, but their exact molecular targets and mechanisms of action in diabetes and its complications require further investigation [9,10,98]. The absorption of each polyphenol in vivo has been evaluated, but the development of optimized dosage regimens will be necessary in the future. Most of the previous research into the pharmacological action of polyphenols has been performed in vitro or using animal models, and a number of advances have been made.

The challenges in clinical application of polyphenols include low bioavailability, rapid metabolism, and the need for strategies such as improved formulation (nanoencapsulation, synergistic combinations, etc.). Further research is needed on polyphenols such as resveratrol, quercetin, and chlorogenic acid, which are suggested to have additive effects with existing antidiabetic and antioxidant drugs.

**Figure 2:**
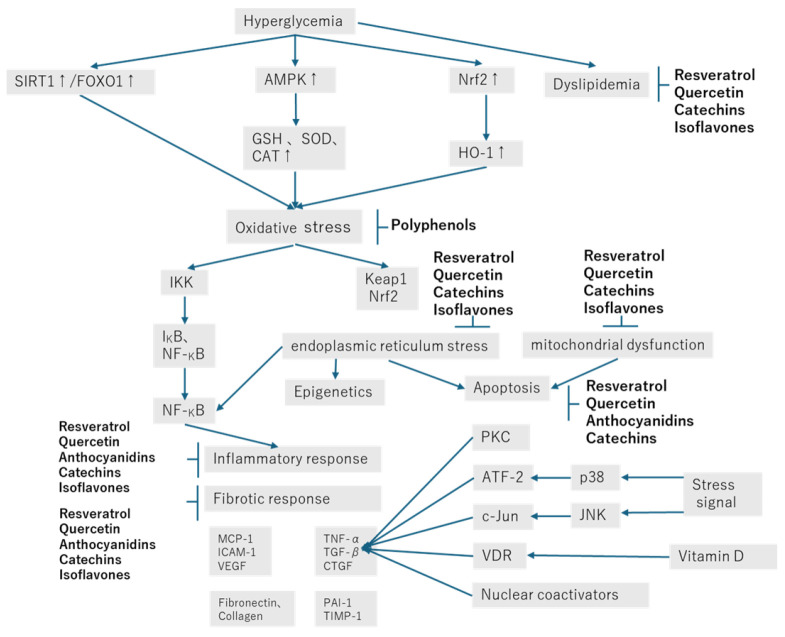
Effects of polyphenols in diabetic nephropathy.

As shown in Figure 2, the effects of polyphenols on diabetic nephropathy can be considered from many angles, but their molecular mechanisms of action, pharmacokinetics, pharmacodynamics, and side effects need to be further investigated through clinical trials. The drugs that are currently used to treat diabetes can be classified as α-glucosidase inhibitors, sulfonylureas, biguanides, and glinides, as well as insulin. In addition, the development of new medicines for the treatment of diabetic complications is an important avenue of current research. To be useful, such drugs should be effective, safe, and inexpensive, and the molecular targets of the active components of plant extracts must be identified. The potential of polyphenols is great, and more studies are likely to be published in the future. Therefore, it seems likely that phenols may be used to limit the progression of diabetic nephropathy in the relatively near future, although it will be necessary to consider the method of administration and the most appropriate amount, according to the stage of the disease.

Finally, the interactions between these polyphenols, specific therapeutic doses, and the long-term safety of these compounds need to be investigated in further studies to optimize their therapeutic utility. Therefore, it is too early to recommend the use of polyphenols as dietary supplements. Despite their therapeutic promise, challenges such as low bioavailability, dose-dependent toxicity, and potential off-target effects require further research to integrate these compounds into clinical practice. In recent years, there have been attempts to integrate the effects of functional foods and other methods to propose comprehensive, sustainable, and personalized nutritional interventions, providing a new perspective for the prevention and management of type 2 diabetes. Future clinical studies are needed to verify the long-term effectiveness and safety of these strategies, and this is an important challenge for the future [99,100].

## Figures and Tables

**Table 1:** The mechanisms of the effects of polyphenols in diabetic nephropathy.

Authors/Year/Reference No.	Polyphenols Used	Model Animals/Cells	Effects
Peng et al. (2019) [19]	Resveratrol and ramipril co-treatment	Rats with streptozotocin-induced diabetes	RhoA/ROCK pathway regulation of early-stage diabetic nephropathy-associated glomerulosclerosis.
Xian et al. (2019) [20]	Resveratrol	Human umbilical cord mesenchymal stem cells in combination with resveratrol to treat non-obese diabetic (NOD) mice	Increased levels of podocyte-associated proteins to better protect renal podocyte function.
Wang et al. (2019) [21]	Resveratrol	Rabbits with diabetic nephropathy and renal failure/renal tubular epithelial (HK-2) cells exposed to high-glucose conditions	Protection against post-contrast acute kidney injury and reduced renal hypoxia, mitochondrial dysfunction, and apoptosis of renal tubular cells.
Xian et al. (2020) [22]	Resveratrol	Non-obese diabetic (NOD) mice	An anti-inflammatory effect and improved renal function by improving metabolic memory of hyperglycemia.
Zhao et al. (2020) [23]	Resveratrol	Rats with streptozotocin-induced diabetes	Protection against diabetic nephropathy through several mechanisms, including improving lipid metabolism and alleviating insulin resistance by inducing autophagy.
Wang et al. (2020) [24]	Resveratrol	*db*/*db* mice	Suppression of oxidative stress-mediated apoptosis of podocytes dependent on 5′ adenosine monophosphate-activated protein kinase (AMPK) activation.
Zhang et al. (2020) [25]	Resveratrol	*db*/*db* mice/renal proximal tubule epithelial (NRK-52E) cells	Protection of renal tubular cells against hyperglycemia-induced apoptosis in diabetic nephropathy by suppressing ER stress.
Gong et al. (2020) [26]	Resveratrol	Lipopolysaccharide-induced rat glomerular mesangial cells	Inhibition of lipopolysaccharide-induced proliferation and inflammation of rat glomerular mesangial cells.
Hashemzaei et al. (2020) [27]	Resveratrol/curcumin/gallic acid	Renal proximal tubule cells	Toxic interactions between mitochondria and lysosomes exacerbated the oxidative stress/cytotoxicity produced by glyoxal. Resveratrol, curcumin, and gallic acid inhibited ROS formation and attenuated glyoxal-induced renal cell death.
Abhaizanjani et al. (2021) [28]	Resveratrol and/or quercetin	Male Wistar rats in hyperglycemic conditions/HEK293 cells	Significant dose-dependent reduction in the amount of methylglyoxal, which had a beneficial effect on aging markers.
Gu et al. (2022) [29]	Resveratrol	C57BL/6J mice fed a high-fat diet for 12 weeks	Reduced lipid deposition in the kidney and improved diabetic nephropathy.
Gu et al. (2022) [30]	Pterostilbene (resveratrol derivative)	C57BL/6J mice fed a high-fat diet for 12 weeks	Alleviation of renal fibrosis and ectopic lipid deposition in the kidneys.
Lan et al. (2023) [31]	Resveratrol	C57BL/6 mice fed galactose for 8 weeks	Alleviation of advanced glycation end product-related renal dysfunction through reduced renal cell senescence, apoptosis, and fibrosis.
Zhu et al. (2023) [17]	Resveratrol	*db*/*db* mice/glomerular mesangial cell line	Prevention of mitochondrial fission attenuated the progression of diabetic nephropathy.
Zhang et al. (2024) [32]	Resveratrol	Human mesangial cells	Suppression of proliferation by suppression of the hyperglycemia-induced miR-1231/IGF1/ERK pathway.
Yan et al. (2024) [33]	Resveratrol	*db*/*db* mice	Amelioration of the progression of diabetic kidney disease by suppression of tubulointerstitial fibrosis, which may be at least partially involved in regulating the gut microbiota–short-chain fatty acid axis.
Lei et al. (2019) [34]	Quercetin	*db*/*db* mice/glomerular mesangial cells	Inhibition of mesangial cell proliferation through reactivation of the Hippo pathway in high-dose glucose-treated mouse glomerular mesangial cells and diabetic nephropathy.
Jiang et al. (2019) [35]	Quercetin	*db*/*db* mice	Alleviation of early diabetic kidney damage by improved lipid metabolism.
Liu et al. (2022) [36]	Quercetin	*db*/*db* mice/mouse podocytes	Attenuation of podocyte apoptosis by inhibition of the EGFR signaling pathway.
Wan et al. (2022) [37]	Quercetin	Human mesangial cells/blood samples collected from diabetic nephropathy patients and healthy controls	Inhibition of HG-induced HMC proliferation, inflammation, and oxidative stress via the miR-485-5p/YAP1 axis.
Feng et al. (2023) [38]	Quercetin	*db*/*db* mice/human renal proximal tubule epithelial (HK-2) cells	Inhibition of ferroptosis in renal tubular epithelial cells by regulation of the Nrf2/HO1 signaling pathway.
Zhang et al. (2024) [39]	Quercetin	*db*/*db* mice/human renal proximal tubule epithelial (HK-2) cells	Inhibition of ferroptosis in renal tubular epithelial cells.
Shahin et al. (2024) [40]	Quercetin and atorvastatin co-treatment	NRK-52E rat kidney epithelial cells	Restoration of cell viability.
Liu et al. (2024) [41]	Quercetin	Mouse model of type 2 diabetes induced by a combination of high-fat diet and streptozotocin (STZ)/human renal tubular epithelial (HK-2) cells	Inhibition of renal tubular epithelial cell apoptosis via the PI3K/AKT pathway.
Guo et al. (2024) [42]	Quercetin and dasatinib co-treatment	*db*/*db* mice/*db*/*db* mice transfected with PPARα or shPPARα overexpression vector	Overexpression of PPARα upregulated the expression of PPARα, which targeted downstream FAO pathway-related proteins, restored renal function, and inhibited renal fibrosis in vitro and in vivo.
Oza et al. (2019) [43]	Formononetin	Rats with type 2 diabetes induced by a high-fat diet and low-dose streptozotocin	Increased expression of SIRT1 in kidney tissue.
Zhang et al. (2019) [44]	Calycosin	*db*/*db* mice/mouse renal tubular epithelial cells	Significant amelioration of diabetes-induced renal inflammation in diabetic nephropathy via inhibition of the NFκB-dependent signaling pathway in vivo and in vitro.
Yang et al. (2020) [45]	Tectorigenin	*db*/*db* mice/human glomerular endothelial cells	Reversal of diabetes-induced glucose and lipid metabolism disorders.
Jheng et al. (2020) [46]	Genistein	KK-Ay mice	Inhibition of the activation of albumin-induced activator protein 1 and the development of reactive oxidative stress, accompanied by a decrease in NADPH oxidase (NOX) gene expression.
Li et al. (2022) [47]	Genistein	Sprague Dawley rats	Inhibition of mesangial matrix expansion and oxidative stress, protected podocyte integrity, and increased mitochondrial membrane potential.
Yosri et al. (2022) [48]	Calycosin	Rats with streptozotocin-induced diabetes	Inhibition of the progression of DN through regulation of the NFκB p65/NLRP3/TXNIP inflammasome signaling pathway.
Chen et al. (2024) [49]	Puerarin	Mice with streptozotocin-induced diabetes	Association with the inhibition of caspase 1-mediated pyrexia.
Li et al. (2024) [50]	Arctigenin and puerarin co-treatment	*db*/*db* mice	Arctigenin and puerarin have an additive inhibitory effect on the activation of the inflammatory NFκB pathway.
Wang et al. (2024) [51]	Puerarin	Renal mesangial cells	Attenuation of hyperglycemia-induced oxidative stress via the RAGE/PKC/NOX4 axis.
Wang et al. (2025) [52]	Puerarin	Mice with streptozotocin-induced diabetes	Regulation of the SIRT1/NLRP3/Caspase 1 pathway to inhibit podocyte pyroptosis, reduce podocyte damage, and alleviate renal inflammatory damage.
Zhu et al. (2020) [53]	(-)-Epigallocatechin gallate	Mice with streptozotocin-induced diabetes	Amelioration of renal fibrosis by targeting Notch via inhibition of the TGFβ/SMAD3 pathway.
Yang et al. (2022) [54]	(-)-Epigallocatechin gallate	Rats with streptozotocin-induced diabetes	Suppression of ER stress-mediated NLRP3 inflammasome overactivation.
Zhang et al. (2024) [15]	(+)-Catechin	Mice with streptozotocin-induced diabetes	Suppression of endoplasmic reticulum stress and NLRP3-related inflammation and reduced renal tubule damage.
Ni et al. (2024) [55]	(+)-Catechin	*db*/*db* mice	Alleviation of diabetic nephropathy through its anti-inflammatory properties and regulation of EMT-related genes, such as *Rage*, *Cav1*, *Grem2*, *Macrod2*, and *Kap*.
